# Was descent in Neolithic and Bronze Age Europe patrilineal or bilateral?

**DOI:** 10.1098/rspb.2025.0815

**Published:** 2025-10-29

**Authors:** Léa Guyon, Evelyne Heyer, Raphaëlle Chaix

**Affiliations:** ^1^ UMR7206 Eco-Anthropologie, CNRS, MNHN, Université Paris Cité, Paris, Île-de-France 75116, France; ^2^ Department of Medicine and Life Sciences, Universitat Pompeu Fabra, Institute of Evolutionary Biology (UPF-CSIC), Barcelona 08003, Spain

**Keywords:** kinship, modelling, ancient DNA, uniparental, patrilineal descent

## Abstract

Many studies have attempted to gain insights into the kinship systems of past human populations using ancient DNA data. Several studies focusing on Neolithic and Bronze Age European sites reported a high male relatedness and a low Y chromosome diversity, and concluded that descent in these past societies was patrilineal and residence was patrilocal. Here, we used simulations to assess male and female relatedness as well as the uniparental genetic diversity expected under different types of descent and residence systems. We confirm that ancient DNA data from most of these sites are compatible with patrilocal residence; however, the claim that many Neolithic and Bronze Age European populations had patrilineal descent is not supported.

## 1. Introduction

Our study aimed to explore the ability of archaeogenetic studies to infer past descent rules from uniparental genetic diversity and sex-specific relatedness. Several studies concluded that residence in many European Neolithic and Bronze Age sites, ranging from 6200 to 1200 before the common era (BCE) and covering a large geographical area, from Eastern and Central Europe [1–8], to Western [9–13], Northern [14–18] and Southern Europe [19–21] was mostly patrilocal and/or that descent was patrilineal (see also Blöcher *et al.* [22] for a Central Eurasian site) (electronic supplementary material, table S1 and figure S1). Some of these studies also used isotopic ratios to assess individuals’ mobility during their lifetime [2,3,7,8], the majority of non-local individuals were females.

In these studies, patrilocal residence means that women move to their male partner’s place, while patrilineal descent is generally used in an undefined sense. In social anthropology, descent is defined as patrilineal if the children belong to the social group (lineage, clan) of the father [23–25]. Such lineages or clans do not exist in bilateral descent systems, in which the household is the largest corporate kin group (electronic supplementary material, figure S2). Hence, most present-day European societies have bilateral descent [23,26]. Inferring the descent rules of past human populations is important not only to understand how families were organized but also to understand how societies were socio-politically and economically regulated. Indeed, in many patrilineal societies, lineages and clans play fundamental socio-political roles, such as managing and transmitting property, organizing religious ceremonies, mediating inter-group relationships and regulating matrimonial unions [24–27]. Unilineal groups are also thought to facilitate collective action because membership to such groups is less ambiguous than in the case of bilateral kindreds, who do not have clear boundaries as they are ego-centred [28,29].

To conclude that descent was patrilineal, these studies relied on the observed high male relatedness and the low Y chromosome diversity often found within these sites. However, it is questionable whether uniparental genetic diversity estimated from archaeogenetic studies, combined with male and female relatedness levels, are informative regarding past descent systems. Indeed, it has been pointed out that a bilateral descent system with a patrilocal residence rule could theoretically result in high relatedness among males, similarly to a patrilineal system [30,31].

To explore the ability of archaeogenetic studies to infer past descent rules from uniparental genetic diversity and sex-specific relatedness, we performed simulations to estimate the levels of male and female relatedness and uniparental genetic diversity expected for patrilineal, bilateral and matrilineal descent systems. We took into account the most frequent residence rules associated with each of these descent rules according to the Standard-Cross Cultural Sample (electronic supplementary material, table S2 [32]): bilateral descent was simulated with patrilocal, matrilocal and ambilocal residence rules, patrilineal descent was simulated with patrilocal residence and matrilineal descent with matrilocal residence. In these simulations, we assumed that individuals are buried either in the cemetery of their village, or in the smaller cemetery of their local group, and we took into account the most likely post-mortem location of females and males under each descent system. To do so, we relied on a study by Ensor *et al.* [33] which compiled ethnographic information of post-mortem locations from the Electronic Human Relations Area Files database (eHRAF) for 28 human cultures spread throughout the world.

In addition, we took into account the diversity of unilineal systems reported by the ethnographical literature. In particular, Barnes has stressed that the patrilineal systems from New Guinea Highlanders, such as the Chimbu and the Huli, contrast with patrilineal systems described in several African populations such as the Tiv and the Tallensi ([34], see also [35–40]). According to Barnes [34], in African patrilineal systems, the patrilineal rule is often observed, the status of descent groups is transmitted intergenerationally on the paternal line (meaning that dominant patrilineal groups remain dominant for several generations), and segmentation of groups is chronic, with lines of cleavage following paternal genealogical lines (a fission type also called lineal) [41]. On the other hand, in New Guinean systems, the patrilineal descent groups comprise a larger proportion of individuals who are not paternally related to each other because the principle of recruitment to the father’s descent group operates concurrently with other principles (such as the residence place of the individual). In addition, the dominance of a group depends on the transitory presence of leaders or ‘big men’ and is not inherited from a generation to another, and segmentations are less common, less predictable and do not follow the paternal genealogical lines (a fission type also called random [41]). Barnes referred to these New Guinean patrilineal systems as systems with weak patrilineal dogma and to African patrilineal systems as systems with stronger patrilineal dogma [34]. Sahlins also emphasized that the extent to which patrilineal populations adhere to the patrilineal rule can vary [30]. Note that the literature also shows that this is not a systematic opposition between New Guinea and Africa, as some New Guinean systems can follow quite strictly the patrilineal rule [42], while some African systems may share features with New Guinean systems [43]. Such variation seems to be related to the economic and ecological conditions of the populations [30,43]. Furthermore, the patrilineal systems of populations from other geographical regions seem to share similarities either with the strict African patrilineal systems (for example, the Turco-Mongols from Central Asia, who appear to follow quite strictly the descent rule [44,45]), or with the looser systems from New Guinean Highlanders (for example, the case of a village in Yemen with high rates of male migration between patrilineal groups [46]). Matrilineal systems have also been reported to follow more or less strictly the matrilineal rule [47–49]. Hence, we believe Barnes’ classification of systems with either weak or strong unilineal dogma (which we call here loose and strict systems) is relevant for the evolution of genetic diversity as these two cases are at the two ends of the theoretical spectrum of unilineal systems, with the loose system cumulating features that prevent the erosion of the uniparental genetic diversity (looseness in group affiliation, random fission and the absence of differential growth between groups), and the strict system cumulating the opposite features that enhance the erosion of the uniparental genetic diversity [50].

Following Barnes’s paper [34], we simulated two variants of the patrilineal system, and by symmetry, we did the same for matrilineal systems (table 1). In the strict unilineal systems, the unilineal dogma is strong, meaning that: (i) individuals are necessarily affiliated with the descent group of their father (for the patrilineal system) or mother (for the matrilineal system), (ii) group fission is lineal, meaning that they follow paternal genealogical lines in the patrilineal case (and maternal genealogical lines in the matrilineal case), and (iii) group membership determines reproductive success, with higher status groups having greater reproductive success. In the loose unilineal system, the unilineal dogma is weak. This implies that: (i) affiliation with the father’s group (patrilineal case) or mother’s group (matrilineal case) is flexible, with horizontal transfers of males (patrilineal case) or females (matrilineal case) from one group to another, (ii) group fission is random, meaning that they do not follow paternal or maternal genealogical lines, and (iii) group identity does not determine reproductive success.

**Table 1.** Comparative table of strict and loose unilineal descent models. For each characteristic of the kinship system, the corresponding parameter in the model is given, along with the corresponding value in the strict unilineal descent system and in the loose unilineal descent system, respectively.

unilineal descent system	kinship system characteristic		corresponding parameter in the model	parameter value
strict	descent group membership of individuals	strictly determined at birth by the unilineal rule	per generation migration rate between villages and descent groups for males (m_m_) in patrilineal systems and for females (m_f_) in matrilineal systems	0
loose		flexible, i.e. may be different from the descent group membership expected given the descent rule, or can change throughout life		0.05 or 0.25
strict	growth rate of descent group	depends on group identity and is transmitted through generations. Some groups have higher social status and higher reproductive rates than others	variance in reproductive success between descent groups σ^2^. In the strict system, the growth rate of each descent group is transmitted (with some variance) to the newly formed groups after fission	0.1
loose		not transmitted through generations. For example, it may depend on the presence in the groups of influential individuals who have acquired their status during life (so-called ‘big men’)		0
strict	descent group segmentation (fission)	lineal fission, i.e. gathering in the new groups individuals that are closely paternally related (patrilineal descent) or closely maternally related (matrilineal descent)	fission type	lineal
loose		random fission, i.e. independently from genealogical lines (e.g. gathering individuals who follow a ‘big man’)		random

## 2. Methods

We conducted simulations by modifying a model established in prior research [50]. To enhance clarity, we provide a recapitulation of the model and the analytical methodologies, along with the modifications implemented to address the specific question of interest in this study.

### (a) Description of the model

**Figure 1.**
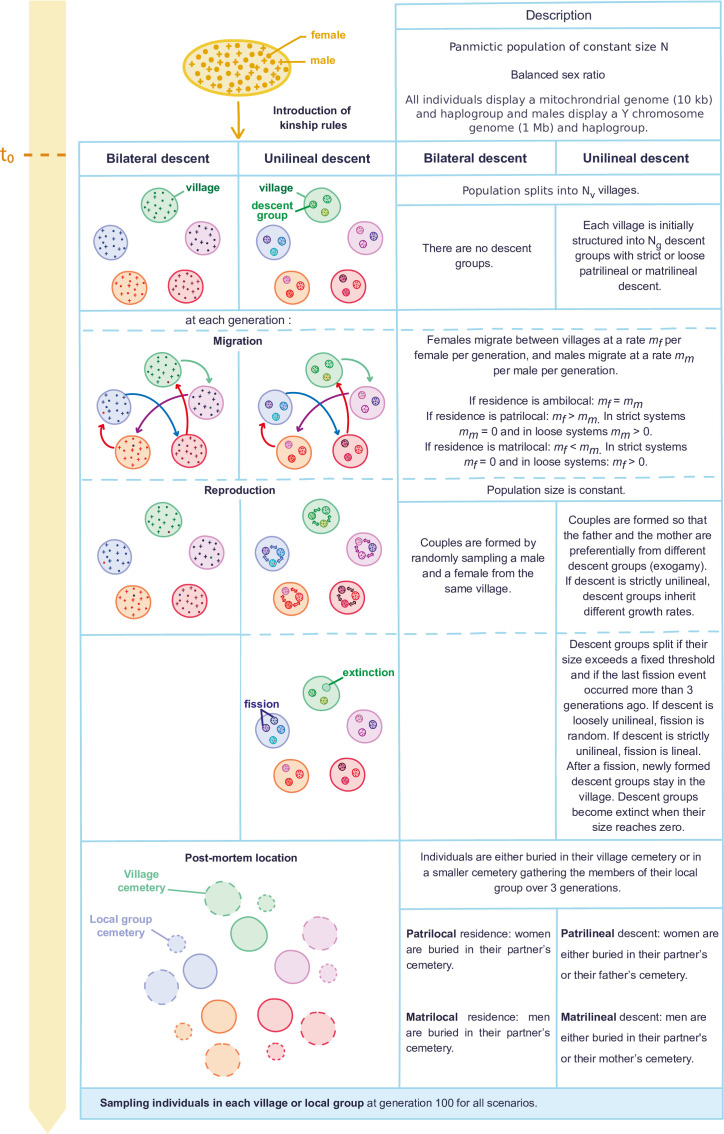
Description of the models. At *t*_0_ = 100 generations ago (after 20 000 generations of panmixia), the kinship rules of interest are introduced. In scenarios with bilateral descent, the population is divided into *N*_v_ villages that are not structured into descent groups. In scenarios with unilineal descent, the population is divided into *N*_v_ villages, themselves divided into patrilineal or matrilineal exogamous descent groups, that experience fission if their size is over *N*_max_ individuals and if the last fission event has occurred more than three generations ago. They can also go extinct if they become empty. In strict patrilineal (or matrilineal) descent scenarios, children are affiliated to their father’s (respectively mother’s) descent group at birth and remain in that group throughout their life, whereas in loose unilineal descent scenarios, they may join another group during their lifetime. Cycles of migration/reproduction/fission and extinction are simulated for 100 generations. Individuals are buried either in the cemetery of their village or in the smaller cemetery of their three generations deep local group (see electronic supplementary material, figure S3 for the definition of local groups according to each descent system). Relatedness and uniparental genetic estimators are computed at the village or local group level. This figure is an adapted version of figure 1 from Guyon *et al*. [50].

We designed a socio-demographic model (figure 1) using SLiM v5 [51], which allows forward simulations of complex socio-demographic models. More precisely, we simulated several scenarios involving a shift from a constant-size panmictic population (*N*_total_ = 1500 or 5000 individuals) to a population structured in either two or five villages, with strictly patrilineal, loosely patrilineal, bilateral, strictly matrilineal or loosely matrilineal descent for 100 generations. In addition, we simulated a two phase scenario with 100 generations of patrilineal descent followed by 100 generations of bilateral descent. All individuals carry mitochondrial DNA (mtDNA) of size 10 kilobases (kb) as well as a mitochondrial haplogroup, and males carry a 1 Megabases (Mb) non-recombining portion of the Y chromosome as well as a Y chromosome haplogroup. To ensure complete coalescence in every simulation, it is advised to simulate a burn-in step for more than 10 times the total size of the population [51]. Therefore, we simulated a panmictic population of constant size *N*_total_ for 20 000 generations (which is higher than 5 × *N*_Y_ when *N*_total_ = 5000 with *N*_Y_ being the effective population size of Y chromosomes and a factor of five because the Y chromosome is haploid) before introducing the kinship rules of interest. All scenarios were simulated with 100 replicates and paralleled using GNU parallel [52].

#### (i) Migration

Following the most frequent residence rules associated with each descent rule (electronic supplementary material, table S2), we simulated a bilateral descent system with patrilocal (either strict or loose), matrilocal (either strict or loose) and ambilocal residence rules, a patrilineal descent system with patrilocal residence (either strict or loose) and a matrilineal descent system with matrilocal residence (either strict or loose). If the residence rule is patrilocal, females migrate more than males between villages. We set the female migration rate at either 10% or 50% per generation (a range corresponding to ethno-demographic estimations in patrilocal populations from Southeast Asia [53] and Central Asia [54]). The male migration rate is set at 0% per generation if the patrilocal residence is strict, and either 5% or 25% if the patrilocal residence is loose (respectively when the female migration rate is 10% or 50%). In these loose systems, each male migrant is simulated to become the adopted son of a randomly picked adoptive father from his new village after he migrated to this new village. Symmetrically, if the residence rule is matrilocal, males migrate more than females between villages, with a male migration rate between villages set to either 10% or 50% per generation. The rate of female migration is set at 0% if the matrilocal residence is strict, and either 5 or 25% if the matrilocal residence is loose (respectively when male migration rate is 10% or 50%). In these loose systems, each female migrant is simulated to become the adopted daughter of a randomly picked adoptive mother from her new village after she migrated to this new village. If the residence rule is ambilocal, as many males as females migrate between villages, with a per generation migration rate of both sex set either to 5 or 25%.

#### (ii) Reproduction

The population size is constant. While descent groups may fluctuate in size, the size of villages is fixed over time (300 in the case of a population of *N*_total_ = 1500 individuals divided in five villages, 750 in the case of a population of *N*_total_ = 1500 individuals divided in two villages and 1000 in the case of a population of *N*_total_ = 5000 individuals divided in five villages, which are compatible with village sizes in the Neolithic period [55]). Mating pairs are monogamous. In the bilateral descent system, offspring result from the reproduction of randomly selected mating pairs within the same village. In the unilineal descent systems, mating pairs are formed by randomly associating a male and a female from two different descent groups within the same village at each generation. Deviations from strict descent group exogamy may occur when a male and a female from different descent groups cannot be mated. In this case, mating pairs are formed by randomly selecting a male and a female from the same descent group, to minimize the number of individuals that remain single. In patrilineal descent systems, children belong to the descent group of their father, whereas in matrilineal descent systems they belong to the descent group of their mother.

In the strict unilineal systems, descent groups display different growth rates. Initially, each descent group i, i∈ [1, 15] is given a growth rate *r*_i_ drawn from a normal distribution of mean *r* = 0 and variance σ^2^ = 0.1. Such variance for both strict patrilineal and strict matrilineal systems was calibrated from patrilineal lines or groups from the ethnographic literature (see Guyon *et al*. [50], tables S1 and S2 for further information). Note that social status applies to both men and women. In strict systems, individuals from high-status groups (both males and females) have more children on average than individuals from low-status groups. In strict patrilineal and patrilocal systems, the status is transmitted intergenerationally on the paternal line. Women from high-status groups may integrate a low-status group upon marriage and *vice versa*. In strict matrilineal and matrilocal systems, the status is transmitted intergenerationally on the maternal line and men can change status and reproductive success upon marriage.

The offspring results from the reproduction of randomly picked mating pairs so that the father’s (resp. mother’s) descent group *j* has a probability *P*_*j*_ given by equation (2.1) to be chosen (with *r*_*j*_ the growth rate of the descent group *j* and *M*_*j*_ the number of males (resp. females) in the descent group *j*) in patrilineal (resp. matrilineal) descent systems.


(2.1) $$P_{j}=\frac{2\times M_{j}\times exp\left(r_{j}\right)}{\sum_{i}^{}2\times M_{i}\times exp\left(r_{i}\right)}$$

In the loose unilineal systems, the growth rates of all descent groups are identical (*r*_i_ = 0).

#### (iii) Fissions and extinctions

A descent group goes extinct if its number of individuals reaches 0. It splits into two new descent groups if its number of individuals exceeds the threshold *N*_max_ = 75 or 150 individuals and if the time between two fission events is above three generations. These threshold values were chosen so that the size of descent groups is compatible with values reported in the literature (see Table 1 in Walker & Hill [56]). The minimal time between two fission events was set to three generations, so that the mean genealogical depth of descent groups stabilizes around 24 generations under the strict patrilineal descent scenario (with *N*_max_ = 150), a value that is consistent with descent group depth in current human populations [45,57–59]. Fissions are lineal in the strict unilineal systems, meaning that they follow unilineal genealogical lines. In other words, in the strict patrilineal system, men who are closely related paternally, that is, who share their own most recent common paternal ancestor (MRCPA) that is younger than the MRCPA of the whole patrilineal parent descent group, stay together in the same new descent group. The proportion of males forming a new group can deviate from 0.5. Symmetrically, in the strict matrilineal system, women who are closely related maternally stay together in the same new descent group. In the loose unilineal systems, fissions are random, meaning that individuals are randomly sampled in the parent descent group to form a new descent group. The proportion of male individuals in each newly born descent group is drawn from a truncated normal distribution with mean 0.5 and variance 0.5, lying within the interval [0,1], which resembles the distribution of proportions of individuals forming a new group in the case of lineal fission. In both systems, once the males (resp. females) are distributed in the two new patrilineal (resp. matrilineal) groups, individuals from the other sex are split proportionally between the two groups so that sex ratio is balanced in the new descent groups. In the strict unilineal systems, growth rates are transmitted from the splitting group to newly formed groups with some variance: new growth rates are assigned to the newly formed groups by drawing them in a normal distribution of mean *r*_*j*_ (the growth rate of the splitting group) and variance σ^2^ = 0.1 at each fission event.

#### (iv) Post-mortem location of individuals

We assumed that individuals are buried either in the cemetery of their village, or in the smaller cemetery of their local group. We took into account the most likely post-mortem location of females and males under each descent system based on cross-cultural observations in contemporary populations [33] as integrated in previous kinship models [60,61]. In bilateral systems, cemeteries usually gather members of the same residence group (including both female and male partners). Most patrilineal societies organize their cemeteries following patrilines, with women buried either with the patrilineal group of their male partner (type P1) or of their father (type P2) [33]. Matrilineal systems organize their cemeteries following matrilines, with men usually buried with their mother [33] (type M2). By symmetry with the patrilineal systems, we also simulated the case where men are buried with their female partner (type M1).

We assumed that local groups are three generations deep. Examples of local groups are shown in electronic supplementary material, figure S3 (assuming each couple has one son and one daughter). More precisely, in the bilateral patrilocal system, the local group cemetery gathers a male focal individual and his female partner three generations ago and their descendants following bilateral descent rule and patrilocal residence rule: their sons and grandsons (if they have not migrated away), along with their female partners. In the patrilineal systems, the local group cemetery gathers a male focal individual three generations ago and his descendants following a patrilineal descent rule: his sons and grandsons (if they have not migrated away). In the P1 system, the female partners of all males are added to the local cemetery group. In the P2 system, the sisters rather than the female partners are added (for all males from the patriline except the male focal individual). In the loose patrilineal systems and the bilateral system with loose patrilocality, local groups also include, if they exist, the adopted son and grandson(s) of the focal male, along with their female partners in the bilateral system and the P1 system, and along with the daughter(s) of the adopted son of the focal male in the P2 system. Local groups in the bilateral matrilocal and matrilineal matrilocal systems were defined symmetrically. In the ambilocal system, local groups consist of a focal individual, his or her partner, their children and grandchildren who have not migrated away, and their partners. One local group cemetery was sampled in each village. All local groups were selected so that they contained at least two males and two females.

### (b) Relatedness coefficients

Relatedness coefficients are computed in SLiM between all pairs of individuals in each village cemetery and each local group cemetery 100 generations after the introduction of the kinship rule of interest for each replicate. SLiM-relatedness coefficients take into account genealogical links between individuals up to three generations in the past. Mean female and male relatedness coefficients were computed for each replicate by averaging the values across villages or across local groups. Mean relatedness coefficients among males and among females were compared within each kinship system at the level of the village cemetery and of the local group cemetery using Wilcoxon signed-rank tests with a Bonferroni correction for *p*-values. Similarly, Wilcoxon signed-rank tests were used to compare mean relatedness among males and among females between kinship systems.

### (c) Haplogroup diversity

Haplogroups are modelled by assigning two unique tags to each individual at the beginning of the simulation. The first tag corresponds to the Y chromosome haplogroup and is directly transmitted from father to son. The second tag corresponds to the mitochondrial haplogroup and is directly transmitted from mother to children. Haplogroup diversity is measured at the level of both the village cemetery and the local group cemetery 100 generations after the introduction of kinship rules by applying the formula in Žegarac *et al*. [62]. Mitochondrial haplogroup diversity is computed from mitochondrial haplogroups carried by females and males. Mean Y chromosome and mtDNA haplogroup diversity was compared within each kinship system at the village and local group levels using Wilcoxon signed-rank tests with Bonferroni correction for *p*-values. Similarly, Wilcoxon signed-rank tests were used to compare mean haplogroup diversity for the Y chromosome and mtDNA between kinship systems.

### (d) Tree management

Every 20 generations from the introduction of descent and post-marital residence rules, sequences of coalescent trees along the genome are output for each modelled genetic marker [51]. These sequences are composed of only one tree since there is no recombination event in Y chromosome and mtDNA. Tree sequences corresponding to Y chromosome and mtDNA are sampled and converted into variant call format files (VCF) with a custom Python script using the package tskit v.0.5.7 [63] and pyslim v.1.0.4. Mutations are generated along the coalescent trees with the Python package msprime v.1.3.1 [64] using a mutation rate of 2.5 × 10^−8^ mutations/nucleotide/generation for the Y chromosome and of 5.5 × 10^−7^ mutations/nucleotide/generation for mtDNA [65,66]. These mutation rates are expressed per generation and are derived from Batini *et al*. [65,66] using a generation time of 25 years. They are within the range of values used in the literature ([5.3−10.3] × 10^−10^ mutations/nucleotide/year for the Y chromosome, and [1.30−2.74] × 10^−8^ mutations/nucleotide/year for mtDNA, according to SI Table 8.2 in Skov *et al.* [67] giving a summary of mutation rates used in the literature). As generations do not overlap in the model, males and females have the same generation time, so the same value of 25 years was used for the Y chromosome and mtDNA. Sampling of individuals and conversion to VCF is achieved by using tskit v.0.5.7.

### (e) Effective population size

In each replicate, 20 individuals (10 males and 10 females) per village were sampled from all villages in order to compute effective population sizes at the level of the village cemetery. Furthermore, one local group was sampled in each village, in each simulation, in order to compute effective sizes at the level of the local group cemetery. The effective population sizes of males and females were estimated using the Nei nucleotide diversity [68] which corresponds to the average pairwise number of differences per site between each pair of sequences within a population. Mean male and female effective population sizes were computed for each replicate by averaging the values across villages or across local groups. Then, male and female effective sizes were obtained by dividing nucleotide diversity with mutation rate (see equations (2.2) and (2.3)).


(2.2) $$N_{e}^{mt}=\frac{\pi^{mt}}{2\times \mu^{mt}}$$

(2.3) $$N_{e}^{Y}=\frac{\pi^{Y}}{2\times \mu^{Y}}$$

Sampling of individuals and estimation of effective population sizes was done 100 generations after the introduction of the kinship rules of interest, and 200 generations in the case of the patrilineal then bilateral scenario. In the latter scenario, we estimated the effective population sizes every 20 generations from the introduction of the kinship rules of interest. Mean male and female effective population sizes were compared within each kinship system at the level of the village cemetery and of the local group cemetery using Wilcoxon signed-rank tests with Bonferroni correction for *p*-values. Similarly, Wilcoxon signed-rank tests were used to compare mean male and female effective sizes between kinship systems.

## 3. Results

### (a) Evidence for patrilocal residence but not patrilineal descent in Neolithic and Bronze Age Europe

Taking into account genealogical links across the past three generations, we estimated the mean relatedness coefficient for females and males in the different kinship systems. In addition, we estimated the genetic diversity for mtDNA (transmitted maternally) and the Y chromosome (transmitted paternally), using two estimators: haplogroup diversity and effective population size (nucleotide diversity normalized by mutation rate).

**Figure 2.**
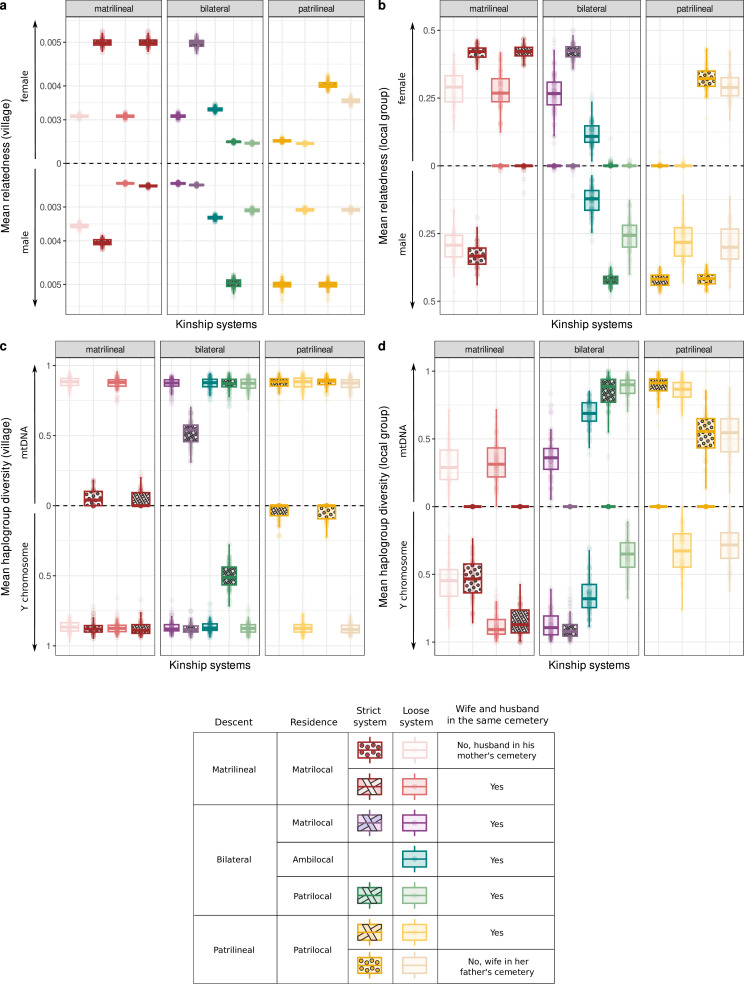
Female and male relatedness and mitochondrial and Y chromosome haplogroup diversity under different kinship systems. Mean relatedness coefficient among females (above the dotted line) and males (below the dotted line) (a,b) and mean mitochondrial haplogroup diversity (above the dotted line) and mean Y chromosome haplogroup diversity (below the dotted line) (c,d) are shown at the level of the village cemetery (a,c) and at the level of the local group cemetery (b,d). Relatedness coefficients and haplogroup diversity are averaged over all villages (a,c) or over sampled local groups (b,d) across 100 replicates. Mitochondrial haplogroup diversity is computed from the mtDNA of females only. The results when mtDNA is sampled in both males and females are presented in electronic supplementary material, figure S4. Each boxplot displays the distribution of averages: the central line within each box indicates the median, the lower edge marks the first quartile and the upper edge marks the third quartile. In panel a, the *y-*axis is displayed using a logarithmic transformation. In this model, the size of the total population was set to 1500 individuals, with villages of size 300 individuals, the migration rate of the dispersing sex was set to 50% per generation (with a migration rate of the philopatric sex of 0% and 25% in the strict and loose systems, respectively) and the descent group fission threshold was set to 150 individuals. Adjusted *p*-values with Bonferroni correction are computed for all comparisons (Wilcoxon signed-rank test) and presented in electronic supplementary material, figure S5.

Figure 2a,b shows levels of relatedness and uniparental diversity expected after 100 generations of the systems described in Methods (see electronic supplementary material, figure S4 for the same figure when sampling both males and females, rather than only females, for the estimation of mitochondrial diversity). In this figure, it is assumed that the size of total population is 1500 individuals with five villages of size 300 individuals, descent groups undergo fission when they grow above a fixed threshold (*n* = 150) and the dispersing sex (females in patrilocal systems and males in matrilocal systems) migrates to another village (and to another group when descent is unilineal) at a per generation migration rate of 50% (calibrated according to Marchi *et al.* [54]). The philopatric sex (females in matrilocal systems and males in patrilocal systems) always stays in its natal village (and group if unilineal), unless the systems are loose, in which case members of this sex migrate to another village (and group if unilineal) at a rate of 25% per generation. In the ambilocal system, 25% of males and 25% of females migrate to another village in each generation.

The results showed that the bilateral descent system with patrilocal residence and the patrilineal descent system with women buried with their male partner’s patrilineal group behave very similarly in terms of expected male and female relatedness: the relatedness among males was significantly higher than among females at the level of the village cemetery and of the local group cemetery (*p*-values ∊ [4.0 x 10^−34^; 6.5 x 10^−25^]). This conclusion is not generalizable to all patrilineal systems: in particular, when the system is loose and women are buried in their father’s village cemetery, the opposite pattern was found (*p*‐value = 8.3 x 10^−32^). The results also showed that no other kinship system produced a similar pattern of high male relatedness and low female relatedness (see *p*-value matrices in electronic supplementary material, figure S5). In addition, we observed that strict patrilineal descent and strict patrilocality in a bilateral system are associated with significantly higher levels of relatedness among males compared with looser systems, at the level of both the village cemetery and the local group cemetery (*p*-values ∊ [8.3 x 10^−32^; 2.6x 10^−22^]).

Furthermore, when looking at haplogroup diversity, patrilineal systems and bilateral systems with patrilocal residence often behaved similarly, with a reduction in Y chromosome diversity compared with mitochondrial diversity (figure 2c,d). No such reduction was observed for systems with matrilocal or ambilocal residence rule. At the level of the local group cemetery, this reduction was always observed (regardless, in the patrilineal case, of the post-mortem location of women) (*p*-values ∊ [1.7 x 10^−36^; 5.0 x 10^−15^]), and was more pronounced for strict patrilineal systems and for the bilateral system with strict patrilocal residence. At the level of the village cemetery, loose systems (with bilateral or patrilineal descent) did not lead to a reduction in Y chromosome diversity compared with mitochondrial diversity (*p*-values = 1.0). At this level, the reduction in Y chromosome haplogroup diversity compared with mitochondrial diversity was only observed for strict systems (*p*-values ∊ [8.9 x 10^−32^; 1.0x 10^−25^]), but was much more severe for the strict patrilineal systems than for the bilateral system with strict patrilocal residence, consistent with Guyon *et al.* [50]. Similar results were found when considering effective population sizes computed from nucleotide diversity (electronic supplementary material, figure S6).

**Figure 3.**
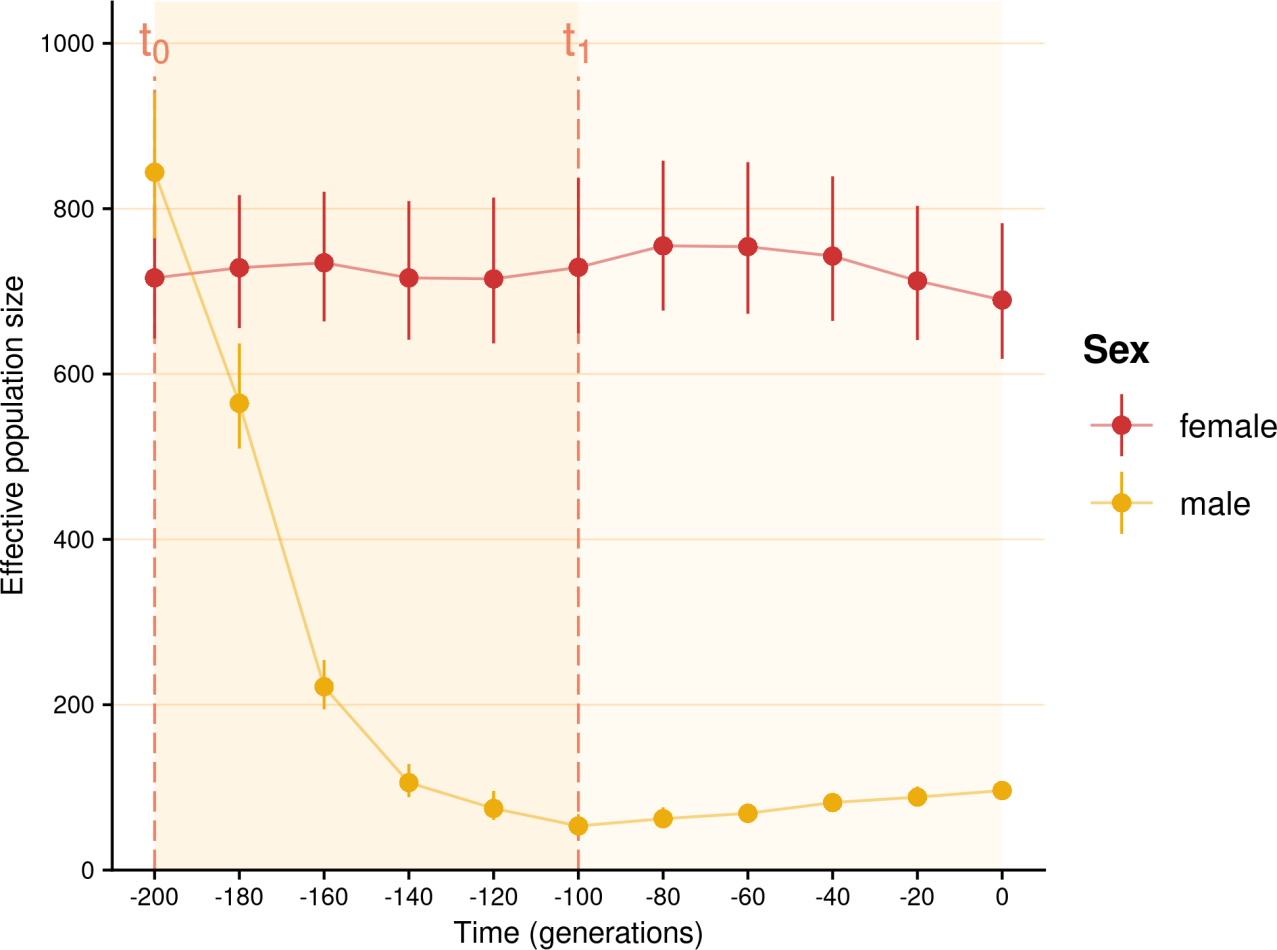
Impact of an ancient patrilineal phase on the effective population sizes of males and females. Male and female effective population sizes over time under a first phase of strict patrilineal system lasting 100 generations (between *t*_0_ and *t*_1_) and a second phase of bilateral system with strict patrilocal residence lasting 100 generations (from *t*_1_). For this figure, sampling was done every 20 generations from the introduction of kinship rules of interest. Each dot represents the mean effective population size across 100 replicates, with error bars indicating the 95% confidence interval around this mean.

Consequently, a strict patrilineal system could theoretically be distinguished from other patrilocal systems by measuring Y chromosome diversity estimators at the village level. However, a strict patrilineal system continues to affect the village Y chromosome genetic diversity for a long time even after being replaced by a bilateral system with a patrilocal residence rule: 100 generations after the end of a strict patrilineal phase, the village male population size was still reduced by 8.8 compared with its initial size (whereas the female population size suffered no reduction) (figure 3). Therefore, if the male ancestors of a population used to be organized according to a strict patrilineal system, the Y chromosome diversity of this population is likely to be low even if this population has shifted to a bilateral system many generations earlier. Thus, the Y chromosome diversity of a population may not be indicative of its prevailing kinship system.

### (b) Disentangling bilateral matrilocal systems from matrilineal matrilocal systems

It is similarly difficult to distinguish a matrilineal system with men buried with their wives from a bilateral system with matrilocal residence using tools presently available to the archaeogenetic field since similar levels of relatedness and haplogroup diversity are expected, except in the case when the whole village mitochondrial diversity can be evaluated. In this case, a strict matrilineal system is expected to lead to a greater reduction in mitochondrial diversity than a bilateral matrilocal system (figure 2, electronic supplementary material, figure S4). However, in most contemporary matrilineal systems, men are returned after their death to their mother’s cemeteries. This provides the opportunity to distinguish such a system from a bilateral matrilocal system, in which both partners are usually buried together [33]. Indeed, in this matrilineal system, adult men are expected to be found in the same cemetery as their mother, male relatedness is expected to be high (figure 2) and when computing mitochondrial diversity by sampling both males and females (electronic supplementary material, figure S4), all of them are expected to carry the same mitochondrial haplogroup while males are expected to carry various Y chromosome haplogroups (see for example, [69]).

### (c) Robustness of conclusions to change in model parameter values

We tested whether our conclusions are robust to changes in the demographic parameters of our model, by evaluating male and female relatedness as well as uniparental diversity expected when making one by one the following changes to our initial model (while holding all other demographic parameters constant): (i) the fission threshold is set to 75 instead of 150 (leading to a total number of fissions of around 185 and extinctions of around 160 in the strict unilineal systems, and of around 35 and around 2 in the loose unilineal systems), (ii) the dispersing sex migrates to another village (and descent group if the system is unilineal) at a rate of 10% per generation while the philopatric sex does not migrate, unless the system is loose, in which case it migrates at a rate of 5% per generation, (iii) the total population size equals 5000 instead of 1500 (meaning that there are 1000 individuals per village), and (iv) the number of villages is set to two instead of five (meaning that there are 750 individuals in each village). The results are shown in electronic supplementary material, figures S7 and S8. Although absolute levels of male and female relatedness and uniparental diversity may vary when making these changes, relative values were conserved. Bilateral systems with patrilocal residence and patrilineal descent systems with females buried in the cemetery of their male partners still behaved similarly in most cases in terms of male and female relatedness and uniparental diversity and stand out from other kinship systems.

## 4. Discussion

Our results show that the significantly higher male than female relatedness and significantly lower Y chromosome than mitochondrial genetic diversity observed in many European Neolithic and Bronze Age sites (electronic supplementary material, table S1) are compatible with a patrilocal residence system, but not with ambilocal or matrilocal residence systems. Our simulations also show that these estimators do not allow to successfully discriminate between patrilineal descent (with women buried with their male partners’ patriline) and bilateral descent (with patrilocal residence), as both systems lead to similar expected genetic signatures and levels of relatedness between males. This is mainly due to the fact that in archaeogenetic studies, the genetic diversity is usually not estimated at the population (or village) level, where we expect different descent rules to differentially affect genetic diversity [50,57,70–75], but at the local cemetery level. In such a setting, the descent rule is not expected to make a difference according to our simulations and to a simple analytical approach proposed by Sahlins 60 years ago [30]. Another reason is that even when diversity can be estimated at the village or population level, an ancient phase of patrilineal descent may produce a long lasting reduction in the Y chromosome diversity. Therefore, it is not possible to tell whether the individuals studied followed patrilineal descent, or whether they were bilaterally organized but their ancestors followed patrilineal descent. Consequently, most European Neolithic and Bronze Age sites are compatible with a patrilineal or with a bilateral descent system. A patrilineal system with women buried with their fathers (a rarer case existing, for example, in the Tallensi [33]) could be distinguished from these two systems as the same difference in uniparental haplogroup diversity is expected, but the female relatedness is expected to be substantially higher. To our knowledge, this genetic pattern has not been observed in archaeogenetic studies so far. Rare evidence for father-to-son inheritance or succession as found in some sites (such as in the Neolithic French monumental site of Fleury-sur-Orne [12]) is often considered as a signature of patrilineal descent, but such transmission is compatible with a patrilineal or a bilateral descent system.

Note that symmetrically, estimates of male and female relatedness and uniparental diversity do not allow to discriminate between matrilineal systems with men buried with their female partner and bilateral systems with matrilocal residence rule. However, because in matrilineal systems, men are often returned to their mother’s cemetery [33], this gives hope to distinguish a matrilineal system from a bilateral matrilocal system from ancient DNA by looking at the burial location of adult men and by evaluating the mitochondrial diversity for both male and female samples [69]. Some archaeological sites, such as the large early Bronze Age Mokrin Necropolis in southeastern Europe, showing high haplogroup diversity for both mitochondrial DNA and Y chromosome [62] are compatible with a wide range of kinship systems, including bilateral systems with matrilocal, patrilocal and ambilocal residence, as well as loose patrilineal and matrilineal systems.

One limitation of our model is that there is a wider range of kinship systems in the ethnographic literature, including many rarer systems, such as systems with bilineal or parallel descent, or with avunculocal, duolocal or neolocal residence (to name a few). Post-mortem location may also be more variable in some populations than described in Ensor’s study [33,76]. Unexpected outcomes such as natural disasters or massacres can also have a major impact on cemetery structures and lessen the effect of kinship systems [77]. In addition, elites and upper social classes are over-represented in ancient DNA studies (e.g. [13,15,78]), which is likely to bias conclusions about the kinship system of the population. Indeed, there may be differential access to burial according to the status of individuals [79], or differential treatment, for example, when warriors were buried under a small burial mound that allowed DNA to be preserved while non-warriors were not [80,81]. Future studies should take into account this larger cultural variation and these potential biases.

Altogether, our results suggest that further investigation is required to decipher descent systems in Neolithic and Bronze Age Europe from archaeogenetic data. While social anthropologists have predicted the existence of unilineal systems in societies of mid-range complexities [82], and while the analysis of modern genomes using coalescent-based methods suggests the existence of such systems around 5000 years ago in different parts of the world [50,83], new estimators should be designed to allow archaeogenetic studies to confirm the existence of these systems during the Neolithic and Bronze Age. So far, the most robust way to discriminate between strict patrilineal segmentary systems and bilateral systems with patrilocal residence is to monitor Y chromosome diversity over time: strict patrilineal segmentary systems are expected to reduce in a fast way the Y chromosome diversity of the population because some patrilineal groups grow at the expense of others owing to their higher social status and reproductive success. This means that, over time, their Y chromosome replaces those from less socially successful groups, leading to a severe decrease in Y chromosome diversity. Such a severe decrease is not expected under bilateral descent with patrilocal residence or under loose patrilineal descent [50]. Evidence for fast replacement of Y chromosomes is starting to accumulate in the archaeogenetic literature, for example in the Corded Ware culture of Bohemia (1900−2400 BCE) with the rapid increase of R1a-M417 haplogroup over 500 years [84], or in the Avar of the Carpathian basin (609−761 AD), with the replacement over a few generations only of the J1a haplogroup with the J2a haplogroup [85]. Early Neolithic communities of Central Europe [8] do not generally show such a sharp reduction in Y chromosome haplogroup diversity (see electronic supplementary material, note S1).

However, at this stage, few other datasets allow a proper monitoring of the Y chromosome diversity over time because the geographical and temporal resolution of samples remains insufficient. Furthermore, the Y chromosome genetic data currently available often lacks resolution because it is obtained using capture methods with low site density on the Y chromosome. We nevertheless believe that it may be a path to explore in the future to infer descent systems. Indeed, palaeogenetic studies have increased the number of ancient samples at numerous neighbouring archaeological sites, and sampling density at these sites is improving. Thanks to the development of targeted capture arrays such as Y-mappable capture assay (YMCA) [86], we can also hope for a better resolution of Y chromosome genetic data. Note that contemporary matrilineal systems are less likely to be segmentary than patrilineal systems (i.e. they are usually organized into clans that rarely split, rather than organized into lineages that chronically split into new sub-lineages with variable growth rates) [30]. But assuming that such strict segmentary matrilineal systems existed in the past, they should lead to a drastic reduction in mitochondrial diversity and monitoring mitochondrial diversity through time might be a way to detect them.

## Data Availability

All data were generated by simulation. Code to run simulations is available at [87]. Supplementary material is available online [88].
